# Dr. W. W. Jaggard

**Published:** 1896-03

**Authors:** 


Dr. W. W. Jaggard, of Chicago, professor of obstetrics in the Chicago Medical College, died January 30, 1896, at Philadelphia, of acute appendicitis, for which an operation was undertaken. For some months previous his health was greatly impaired, following upon the death of his wife. The *Medical Recorder* in writing of the sad event says :

> The termination of his life ends the career of a man of great brilliancy of intellect and extraordinary teaching powers. Every one who came in contact with Dr. Jaggard recognised at once his mental as well as his physical forcefulness, and his control of people and patients was most remarkable. He was educated at the University of Pennsylvania, spent some time in the naval service of the United States and after coming to Chicago became identified with the Chicago Medical College and a number of hospitals. He was forty years of age and leaves one child.

